# Prediction of the Secretome and the Surfaceome: A Strategy to Decipher the Crosstalk between Adipose Tissue and Muscle during Fetal Growth

**DOI:** 10.3390/ijms21124375

**Published:** 2020-06-19

**Authors:** Muriel Bonnet, Nicolas Kaspric, Kimberly Vonnahme, Didier Viala, Christophe Chambon, Brigitte Picard

**Affiliations:** 1 INRAE, Université Clermont Auvergne, VetAgro Sup, UMR Herbivores, F-63122 Saint-Genès-Champanelle, France; kaspric.nicolas@gmail.com (N.K.); brigitte.picard@inrae.fr (B.P.); 2 Department of Animal Sciences, North Dakota State University, Fargo, ND 58108, USA; kimberly.vonnahme@zoetis.com; 3 INRAE, PFEMcp, QuaPA UR 370, F-63122 Saint Genès Champanelle, France; didier.viala@inrae.fr (D.V.); christophe.chambon@inrae.fr (C.C.)

**Keywords:** proteome, secretome, surfaceome, fetal physiology, adipose tissue, muscle

## Abstract

Crosstalk between adipose and muscular tissues is hypothesized to regulate the number of muscular and adipose cells during fetal growth, with post-natal consequences on lean and fat masses. Such crosstalk largely remains, however, to be described. We hypothesized that a characterization of the proteomes of adipose and muscular tissues from bovine fetuses may enhance the understanding of the crosstalk between these tissues through the prediction of their secretomes and surfaceomes. Proteomic experiments have identified 751 and 514 proteins in fetal adipose tissue and muscle. These are mainly involved in the regulation of cell proliferation or differentiation, but also in pathways such as apoptosis, Wnt signalling, or cytokine-mediated signalling. Of the identified proteins, 51 adipokines, 11 myokines, and 37 adipomyokines were predicted, together with 26 adipose and 13 muscular cell surface proteins. Analysis of protein–protein interactions suggested 13 links between secreted and cell surface proteins that may contribute to the adipose–muscular crosstalk. Of these, an interaction between the adipokine plasminogen and the muscular cell surface alpha-enolase may regulate the fetal myogenesis. The in silico secretome and surfaceome analyzed herein exemplify a powerful strategy to enhance the elucidation of the crosstalk between cell types or tissues.

## 1. Introduction

An adequate development of adipose tissue (AT) relative to muscle (i.e., lean-to-fat ratio) is central both for issues related to the human health and to the meat industry. Indeed, excessive fat accumulation is related to human obesity and related pathologies, such as metabolic syndrome, insulin resistance, or cardiovascular diseases. Moreover, fat and muscle masses within the bodies of species of agronomic interest are among the major determinants of carcass economic value in the meat industry [1,2]. The lean-to-fat ratio is the result of a dynamic balance between the number and volume of muscular and adipose cells, respectively. The fetal life is the unique period during which the muscular masses grow, both by an increase in the number of cells (hyperplasia) and by cell enlargement (hypertrophy) in large mammals, such as humans and bovines. Indeed, the total number of muscle fibers is set by the end of the second trimester of gestation in bovines [3] and in humans [4]. This results from the proliferation and differentiation of the successive generations of myoblasts. Conversely, the number of adipocytes is set by birth or by early adulthood, depending on the anatomical location of the AT in both humans [5] and bovines [1]. Thus, the fetal life is a key period of study to understand the mechanisms underlying the dynamic balance between the number and volume of muscular and adipose cells in large mammals. However, this implies knowledge of the cellular and molecular events underpinning the fetal growth of AT and muscle. In muscles, the molecular pattern contributing to the proliferation and differentiation of the different successive generations of myoblasts has been studied using genomic tools [6,7,8,9]. A proteomic analysis of the *Semitendinosus* muscle from Charolais fetuses allowed a mapping of 296 proteins with abundances modified across the fetal life [6,7,8,9]. High modifications of the muscle proteome were observed mainly during the two first trimesters of gestation, with changes in the expression of genes regulating proliferation and the induction of differentiation. A balance between proliferation and apoptosis controlling the total number of fibers has been evidenced [6,7,8,9]. The main modifications of the muscle proteome during the last trimester of fetal life were complex modifications in proportions of protein isoforms that govern contractile and metabolic differentiation [6,7,8,9]. In AT, the total number of perirenal adipocytes increases strongly until the end of the fetal life in bovine [10,11]. The volume of adipocytes increases mainly during the last 40 days of pregnancy in bovines from lean breeds [11] while this occurs earlier (110 to 40 days before birth) in bovines from fat breeds. However, the molecular patterns underlying these cellular events of tissue growth are scarce in mammals [1]. A unique proteomic study has identified 143 proteins in the bovine fetal AT, some of which were proposed to regulate adipocyte precursor proliferation by controlling cell cycle progression and apoptosis or delaying peroxisome proliferator-activated receptor gamma (PPAR)-induced differentiation [11], a master pathway of adipogenesis. A first attempt to understand the growth of muscle relative to AT using available data [12] was limited by the low number of proteins identified as putatively secreted and the low number of proteins identified by proteomics. Thus, an in depth exploration of both adipose and muscular proteomes during fetal life is of critical importance for an improved understanding of the mechanisms underlying the dynamic balance between muscle and AT growth. This balance may occur through tissue-specific signalling pathways and through the secretion of proteins by AT (adipokines) and skeletal muscle (myokines), putatively regulating their growth in a reciprocal manner. The mechanisms underlying the “adipose–muscular” crosstalk are, however, poorly understood [1,13], and remain to be deciphered during fetal development.

The main objective of the present study was to characterize fetal adipose and muscular proteomes in order to identify proteins that may sustain an “adipose–muscular” crosstalk, and thus contribute to the regulation of the balance between muscle and AT growth. The fetal age of 140 days post-conception (dpc) was chosen because both adipose and muscular tissues grow by hyperplasia and hypertrophy [1]. Proteomics data were mined to identify the secretome of fetal adipose and muscular tissues using the web service ProteINSIDE, dedicated to the analysis of ruminant omics data [12]. Moreover, the proteomics data were analyzed relative to the known cell surface subproteomes or surfaceomes in eukaryotes [14,15,16], and were mined using the BUSCA web service [17] in order to identify proteins that could be involved in the “adipose–muscular” crosstalk. The original insights provided by both the proteomics data and the data mining methods constitute a starting point in our understanding of the growth of muscle relative to the growth of AT.

## 2. Results

### 2.1. Proteome Description of the Fetal Bovine Muscle and Adipose Tissues

**Figure 1:**
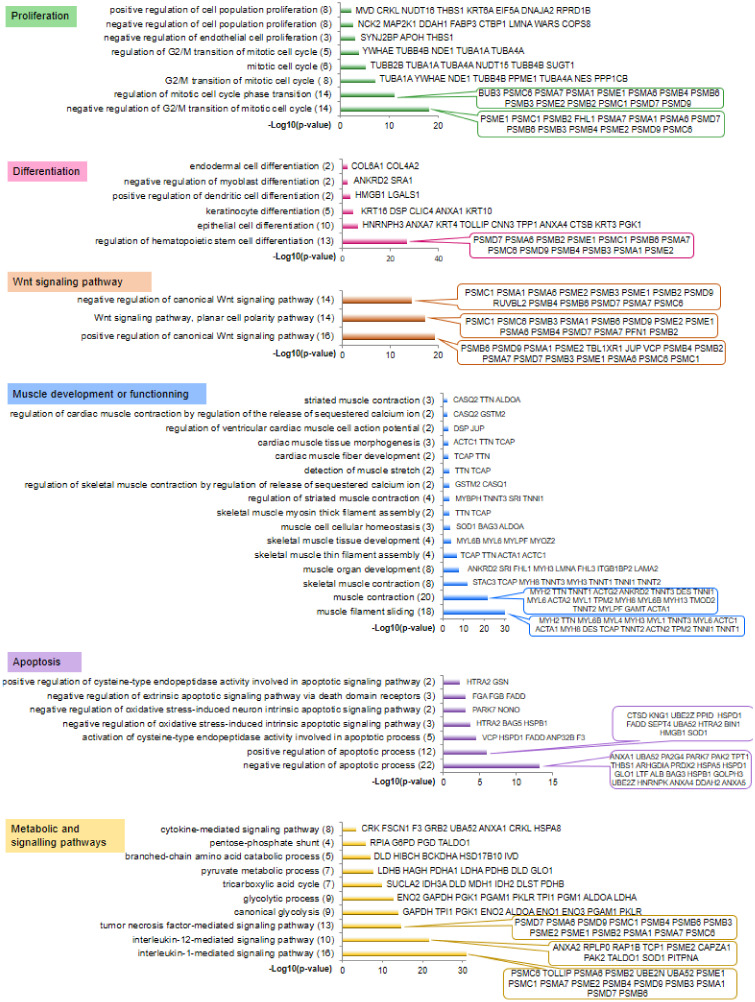
A focus on the most significantly enriched Gene Ontology (GO) terms that annotated proteins identified in the fetal muscles (both *Semitendinosus* and *Longissimus thoracis* muscles) at 140 days post-conception. GO terms were grouped by major biological functions. Results were obtained with ProteINSIDE and enrichments are expressed as −log_10_ (*p*-value) for visualization on graphs, which means that log_10_ values of (*p*-value) of 3, 2, and 1.3 correspond to *p*-values of 0.001, 0.01, and 0.05, respectively. The total number of proteins annotated by GO terms is shown in brackets.

**Figure 2:**
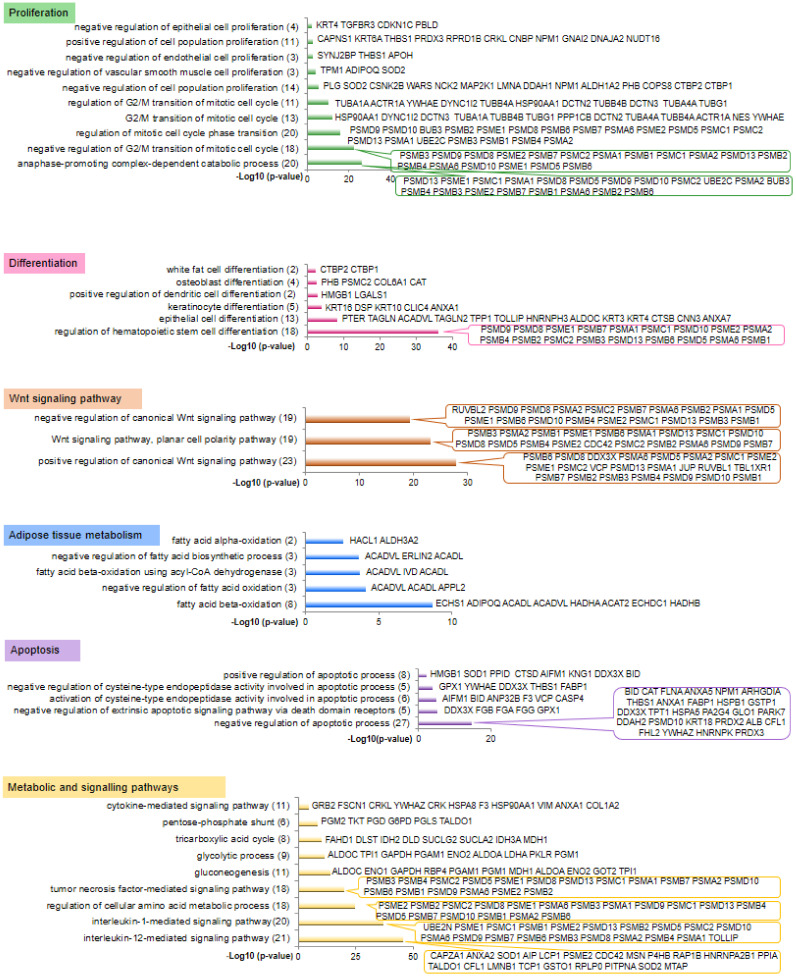
A focus on the most significantly GO terms that have annotated proteins identified in fetal adipose tissue (both perirenal and omental adipose tissues) at 140 days post-conception. GO terms were grouped according to major biological functions. Results were obtained with ProteINSIDE and enrichments are expressed as −log_10_ (*p*-value) to visualized on graphs, which means that log_10_ values (*p*-value) of 3, 2, and 1.3 correspond to *p*-values of 0.001, 0.01, and 0.05, respectively. The total number of proteins annotated by GO terms is shown in brackets.

A total of 751 bovine proteins were identified in both perirenal and omental AT (61% of the proteins had a differential (*p* < 0.05 one-factor ANOVA) abundance between the two AT), while 514 proteins were identified in *Semitendinosus* and *Longissimus thoracis* muscles (42% of the proteins had a differential abundance between the two muscles). Of these, 704 and 490 bovine proteins from AT and muscles were assigned to human orthologs (Supplementary Table S1). The human orthologs were subjected to a functional annotation according to Gene Ontology (GO) analysis. Numerous enriched (*p* < 0.01) GO terms related to biological processes were found for both muscle (Figure 1) and AT (Figure 2) as a consequence of 331 proteins identified in both AT and muscle. The GO terms that annotated both adipose and muscular proteins were grouped into five main functional categories: cell proliferation, cell differentiation, Wnt signalling, apoptosis, and metabolic or signalling pathways.

GO terms related to cell differentiation have annotated 34 muscular (Figure 1) and 42 adipose (Figure 2) proteins, among which 25 proteins were identified in both tissues. Of the proteins specifically identified in muscle, ANKRD2 and SRA1 were annotated by the GO term “negative regulation of myoblast differentiation”. Of the adipose-specific proteins, CTBP2 and CTBP1 were annotated by the GO term “white fat cell differentiation”. Moreover, proteins found in AT (PHB, PSMC2, CAT) or in both AT and muscle (COL6A1) were annotated by the GO term “osteoblast differentiation”, which is consistent with the balance of stem cell differentiation between the adipose, muscular, and bone cell lineages.

GO terms related to the Wnt signalling pathway annotated 18 muscular (Figure 1) and 25 adipose (Figure 2) proteins, among which 14 proteins were identified in both tissues. Most of the muscular and AT proteins were of the proteasome complexes and were annotated by GO terms relative to both the positive and negative regulation of the Wnt signalling pathway. An additional protein, junction plakoglobin (JUP), identified in the AT and muscle, was annotated by a GO term relative to the positive regulation of the Wnt signalling pathway. CDC42 identified in AT was reported to be involved in adipose stem cell induction and differentiation through Wnt/β-Catenin signalling.

Apoptosis was one the most annotated biological processes in both the fetal muscular (40 proteins, Figure 1) and adipose (42 proteins, of which 24 were also identified in muscle, Figure 2) proteomes. In both muscle and AT, most of the proteins would be involved in the negative regulation of the apoptotic process, since 22 muscular and 27 adipose proteins were annotated with related GO terms. Only 12 muscular and 8 adipose proteins were annotated by the GO term for positive regulation of the apoptotic process. Of the muscular proteins, HTRA2 and PAK2 are known to be involved in the regulation of cell death and cell survival; while AIFM1, FHL2, and NPM1, identified in AT, are well-known regulators of apoptosis.

Other enriched GO terms grouped in the metabolic and signaling pathway category annotated 61 muscular (Figure 1) and 76 adipose (Figure 2) proteins, among which 44 proteins were identified in both tissues. The enriched GO terms highlighted pathways known to sustain tissue growth, such as interleukin-1- and interleukin-12-mediated signalling pathways, tumor necrosis factor-mediated signalling pathway, canonical glycolysis, tricarboxylic acid cycle, branched-chain amino acid catabolic process, pentose-phosphate shunt, and the cytokine-mediated signalling pathway.

The proteins that were specifically identified in the fetal muscles (159 human orthologs) and AT (373 human orthologs) were annotated by GO terms related to specialized functions of muscular and adipose tissues. Within the muscle dataset, several GO terms were grouped in the functional category “muscle development and functioning”, which annotated 45 proteins (Figure 1) with GO terms mainly related to muscle contraction, organization, or development; to the assembly of actin and myosin filaments; and in the acquisition of muscle contraction function. Within the adipose dataset, some enriched GO terms were grouped within the functional category “lipid metabolism”, which annotated 13 proteins (Figure 2) mainly involved in the regulation of the balance between fatty acid synthesis and oxidation. Of the adipose proteins, ERLIN2 is known to be involved in the lipid metabolism through a regulation of the SREBP signalling pathway.

### 2.2. Prediction of Proteins Secreted by the Fetal Bovine Muscles and Adipose Tissues

In order to identify the most complete secretome, computational predictions were realized using the bovine sequences of proteins identified in the present study, supplemented by those previously identified in bovine adipose tissue [11] and muscle [6,7,8,9]. Thus, the sequences of 750 adipose and 531 muscular proteins were used for secretome prediction. Among these proteins, ProteINSIDE predicted that 38 and 68 proteins are secreted due to a signal peptide by the fetal muscle and AT, with 29 proteins found to be secreted both by AT and muscle (Table 1).

Because some proteins are secreted through cellular pathways that do not involve a signal peptide, ProteINSIDE predicted proteins that may be secreted either by endosomal recycling, plasma membrane transporter, membrane flip-flop, or membrane blebbing, which involves formation of vesicles or exosomes. As the merged results of the 3 methods of prediction (UniProt subcellular location, a GO term related to a secretory function; and TargetP prediction), ProteINSIDE proposed 10 and 20 additional proteins potentially secreted by the muscle and the AT, respectively, with 8 of these proteins being secreted by both tissues (Table 2). Thus, 48 muscular and 88 adipose proteins were predicted to be secreted by fetal bovine tissues, with 46 muscular and 82 adipose proteins of these also listed among the human [18] or bovine [19] plasma proteins, or as bovine adipokines, myokines, or adipomyokines [20]. Among the proteins that we proposed as being secreted, the muscular SEPT4 and MESD proteins, as well as the adipose MESD, CKAP4, SEPT5, APPL2, STAM, and LAMTOR1 proteins, were only identified in the present study.

### 2.3. Prediction of Fetal Bovine Muscle and Adipose Tissue Surfaceomes

**Figure 3:**
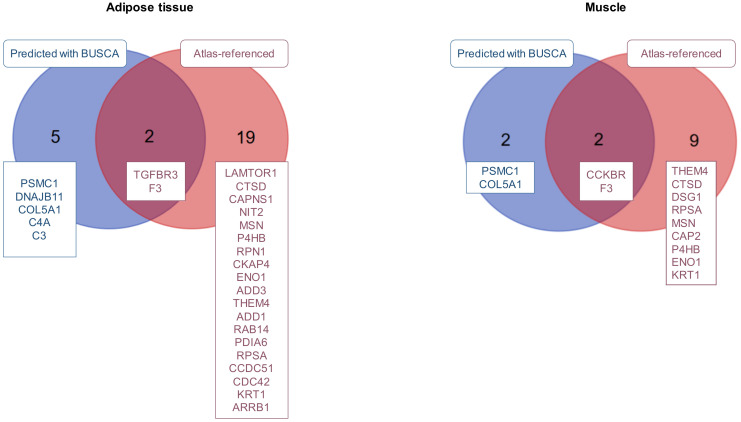
Proteins proposed to be present at the cell surface, and thus to contribute to the surfaceome of bovine fetal adipose tissue and muscle. Putative cell surface proteins were identified either by prediction using the BUSCA web server or by comparison between the lists of fetal bovine proteins and the list of 5956 proteins referenced at the cell surface by merging results from [14,15,16] and the named surfaceome atlas. The Venn diagrams (http://bioinformatics.psb.ugent.be/webtools/Venn/, VIB/UGent, Bioinformatics & Evolutionary Genomics, Gent, Belgium) of the fetal bovine adipose tissue and muscle proteomes show the intersection between the prediction and the comparison with the surfaceome atlas.

The sequences of 750 adipose and 531 muscular proteins were also used for surfaceome prediction. From the bovine protein sequences, the BUSCA web server predicted 7 adipose and 4 muscular proteins present at the cell surface, 2 of which were (PSMC1 and F3) identified in both tissues (Figure 3). When gene names of the fetal bovine proteins were compared to the atlas of plasma membrane proteins predicted in eukaryotic species, 21 adipose and 11 muscular bovine proteins were listed at the intersection with the atlas. As the result of the union between the list of proteins predicted by the BUSCA web server and the atlas, we proposed 26 adipose and 13 muscular proteins identified in bovine fetal tissues (Figure 3), as putatively located on the cell plasma membrane.

### 2.4. Predictions of Proteins Interactions between Adipose Tissues and Muscles

**Figure 4:**
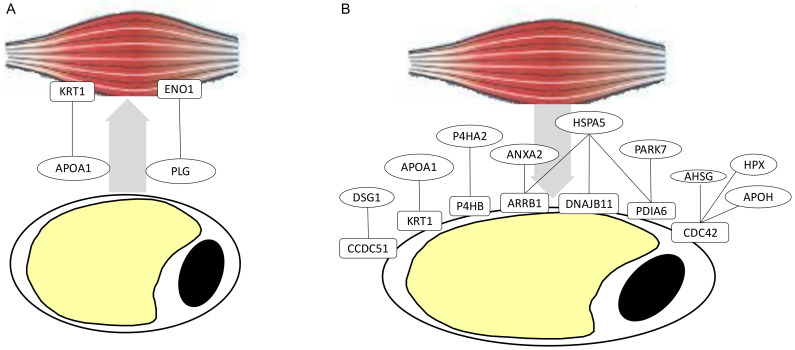
Network of interacting proteins between the proteins potentially secreted by (**A**) Adipose and the muscular cell surface proteins, or (**B**) between the muscle and adipose cell surface proteins. Protein–protein interactions (PPI) between the proteins were identified thank to the PPIs referenced within UniProt, IntAct, and BioGrid databases, which were schematized between an adipocyte and muscle cells.

In order to understand the “adipose–muscular” crosstalk, known protein–protein interactions (PPI) between proteins secreted by one tissue and proteins present at the cell surface of the other tissue were searched. Regarding proteins secreted by AT that may interact with muscular cell surface proteins, 2 PPI between 4 distinct proteins were identified (Figure 4A). The identified PPI linked APOA1 and PLG secreted by AT with KRT1 and ENO1 located at the cell surface of muscle cells.

Regarding proteins secreted by muscles that may interact with cell surface proteins of the adipose cells, 11 PPI between 16 proteins were identified (Figure 4B). The secreted muscular P4HA2, HSPA5, ANXA2, HPX, APOH, AHSG, PARK7, DSG1, and APOA1 are known to interact with the following adipose cell surface proteins P4HB, KRT1, ARRB1, DNAJB11, PDIA6, CDC42, and CCDC51.

## 3. Discussion

This study is the first to establish an enriched proteome of *Semitendinosus* and *Longissimus thoracis* muscles as well as perirenal and omental ATs for the same fetuses. It provides an accurate description of the main functional pathways and biological functions involved in myogenesis, adipogenesis, as well as the first description of the secretome and surfaceome as a prerequisite to understand the crosstalk between fetal adipose and muscular tissues. The most novel results concern the prediction of secreted and cell surface proteins. Indeed, 51 putative adipokines, 11 myokines, 37 adipomyokines, and 26 adipose and 13 muscular cell surface proteins were predicted, and thus may contribute to the crosstalk between AT and muscles during bovine fetal growth.

### 3.1. Pathways that Sustain Fetal Bovine Adipogenesis and Myogenesis

Regarding previously published proteomes of fetal perirenal AT [11] or *Semitendinosus* muscle [7,8], the data mining of the present fetal proteomes has confirmed pathways important for myogenesis and adipogenesis, highlighting three new functional pathways as being important for myogenesis and adipogenesis in bovines, namely apoptosis, Wnt signalling, and cytokine-mediated pathways. Of the confirmed pathways, proliferation, differentiation, muscle development (structural organization and contraction), and lipid metabolism in AT were obviously highlighted by GO terms that have annotated several proteins, with some of them being described for the first time in fetal bovine tissues, such as the carboxy-terminal-binding proteins 1 and 2 (CTBP2 and CTBP1). These proteins belonging to CTBP family are involved in numerous biological processes through their association with several transcription factors. CTBP1 was shown to be able to fix onto the promoter of myogenin [21], a master myogenic regulatory factor, and in this way CTBP1 may regulate the differentiation of bovine fetal muscular cells. CTBP1 was previously identified in fetal AT [11], and the present identification of CTBP2 suggests that both isoforms (CTBP1 and CTBP2) may contribute to the differentiation of adipose precursor cells, either to the white or brown adipocyte lineages [22]. Interestingly, other newly identified proteins were subunits of the proteasome, which were more numerous in AT than muscle (15 proteins in AT and 9 in muscle). This may be related to the involvement of the proteosome in the inhibition of adipogenesis by the degradation of ubiquitinated adipogenic transcription factors, namely peroxisome proliferator-activated receptor gamma (PPAR-γ) [23] and CCAAT/enhancer-binding protein alpha (CEBP-α) [24]. In bovines, as in other mammals, a fine balance between autophagy and ubiquitination could be the determinant for the balance between proliferation and differentiation by modulating the abundance of the master gene of adipogenesis PPAR-γ. In agreement with this hypothesis, some of the new proteins identified were described to be involved in the autophagy process (CTSD, HMGB1, and ANXA7 [25,26]), especially in its positive regulation (PAFAH1B2 and SH3GLB1 [27,28]), along with the formation of autophagosome necessary for the macroautophagy process (RAB1A [29]). These results reinforced our previous hypothesis of an engagement of autophagic proteins to the processes of adipogenesis and myogenesis [12]. Basically, both pathways and proteins identified by the present in-depth proteome characterization are consistent with our knowledge of myogenesis and adipogenesis for 140-day-old bovine fetuses. At 140 dpc (half of the fetal age), myogenesis is characterized by the proliferation of the second and third generations of myoblasts and by the differentiation of the myotubes from the different generations, but mainly from the first generation [3,30]. At 140 dpc, the pre-adipocyte cells proliferate and differentiate in adipocytes [1].

Of the new pathways identified by GO analysis that may contribute to the bovine adipogenesis and myogenesis, the present study has identified proteins related to apoptosis, Wnt signalling, and cytokine-mediated pathways. A large number of GO terms were related to apoptosis, mainly to the negative regulation of apoptosis. These data are in agreement with the results from bovine adult *Longissimus dorsi* muscle [31], as well as from the fetal *Semitendinosus* muscle [7], revealing a fine balance between cell proliferation and apoptosis during the two first trimesters of gestation in this bovine muscle, leading to the control of the total number of fibers. This massive negative regulation could be necessary to prioritize cell proliferation, as already observed in guinea pigs [32]. Among the proteins annotated by GO terms related to the negative regulation of apoptosis, we have identified GLO1, HSPA5, and TPT1 in both AT and muscle. The protein GLO1 (lactoylglutathione lyase, or glyoxalase I), which is the natural defense against dicarbonyl stress (through the detoxification of methylglyoxal, a metabolite of glucose, protein, and fatty acid degradation pathways), was shown to contribute to the glucose uptake [33] and collagen homeostasis [34] in L6 myoblasts. GLO1 was identified in adipose-derived mesenchymal stem cells isolated from mice [35], however its role in adipogenesis or myogenesis remains largely unknown. While the tumor suppressor p53 (TPT1) is a key component in the induction of cell cycle arrest, apoptosis, and DNA repair in response to various stress stimuli, the role of cellular tumor antigen p53 (Tp53) in cellular metabolism is gradually emerging [36]. Of the Tp53 roles specifically in adipocytes and myoblasts, Tp53 was shown as a negative regulator of in vitro adipogenesis in monogastrics [37], while Tp53 was shown to be a master regulator of myoblast proliferation and apoptosis in avian myogenic cell lines [36]. HSPA5 (or GPR78) encodes a member of the heat shock protein 70 (HSP70) largely described to regulate apoptosis. The precise role of HSPA5 during myogenesis remains to be elucidated, however an anti-apoptotic role of HSP70 was proposed in skeletal muscle by Gao et al. [38]. They reported a regulation of TNF-α-induced cell apoptosis through the HSP70/CHIP/ASK1 complex, in which Hsp70 promotes ASK1 proteasomal degradation and prevents TNF-α-induced cell apoptosis. Hsp70 proteins are also known to sequester pro-apoptotic factors, such as BCL-2 [39]. HSPA5 was shown to be essential to stimulate adipocyte differentiation in vitro [40] through an interaction with a member of the K+ channel tetramerization domain family (KCTD15), implicated in crucial physiopathological processes [41].

In the present study, the Wnt pathway has been highlighted in both AT and muscle, in agreement with the activation of Wnt/β-catenin signalling known to promote differentiation of mesenchymal precursor cells into myocytes, while suppressing commitment to the adipocytic lineage and terminal differentiation. Wnt signalling represses adipocyte differentiation by inhibiting the expression of PPAR and CEBP, two central regulators of adipogenesis [42]. Conversely, Wnt signalling stabilizes β-catenin and increases the expression of myogenic factors (pax 3, MyoD) [43]. The present study has revealed proteins as potential targets or partners of the Wnt signalling in the regulation of adipogenesis or myogenesis, such as the proteasome subunits already known to be involved in the PPAR-induced degradation of β-catenin to allow preadipocyte differentiation [44]. The cell division control protein 42 homolog protein (Cdc42) has been identified in the bovine fetal AT, which is consistent with the recently reported involvement of Cdc42 in the stimulation of the proliferation of adipose-derived mesenchymal stem cells [45]. The F-box-like and WD-repeat-containing protein TBL1XR1, which is essential for transcriptional repression mediated by the unliganded nuclear receptor, has been identified in both AT and muscle, and was described to interact with β-catenin [46]. However, its roles in myogenesis and adipogenesis still remain to be determined.

Other proteins were annotated by GO terms related to the cytokine-mediated signalling pathway, which is consistent with the large number of cytokines related to the post-natal muscular [20] and AT growth in bovines [20,47,48]. Of these proteins, growth factor receptor-bound protein 2 (GRB2) is an adapter protein that provides a critical link between cell surface growth factor receptors and the downstream signalling pathways. In murine muscle, GRB2 can be a downstream effector of the hepatocyte growth factor required for the development of secondary fibers [49]. In AT, GRB2 is a downstream effector of the insulin-like growth factor 1 signalling that is known to lead to either proliferation or differentiation of the adipose progenitors [50]. The identification of GRB2 as a central protein within the total PPI network for both AT and muscle is consistent with the contribution of the second generation of fibers to muscle growth at 140 dpc and to the balance between proliferation and differentiation for AT growth.

### 3.2. Secretome and Surfaceome Involvement in Deciphering the Adipose and Musclar Crosstalk

The most original contribution of the present study is the identification of putatively secreted and cell surface proteins, paving the way for the understanding of their interactions, and thus the crosstalk between AT and muscle. Bidirectional crosstalk between adipose and muscular tissues plays important roles in determining body composition. This crosstalk is highly suggested by a competition between the growth of AT and muscle and is not yet fully understood [1]. It probably includes the release of paracrine or endocrine factors, direct cell–cell interactions, and cell interactions with the extracellular matrix. From the present data, we confirmed that 12 proteins (HSP90B1, ADIPOQ, AFP, APOA1, APOA2, COL6A2, ERLIN2, FGG, P4HB, PDIA3, SERPINA1, and TTR) could be secreted by both AT and muscle, as previously proposed in fetal perirenal AT and *Semitendinosus* muscle [12]. Our study highlighted 42 new proteins potentially secreted by the muscle. Some of these proteins act as autocrine or paracrine factors to regulate pathways that we have discussed to sustain myogenesis, including: regulation of cell proliferation (OGN [51], RBP4 and MAP2K1 [52]), regulation of apoptosis (APOH [53], KNG1 [54], and LGALS1 [55]), or muscle development (LAMA2 [56]). Our study highlighted 76 new proteins potentially secreted by the fetal AT. These could be involved through autocrine or paracrine effects in the regulation of apoptosis (plasminogen, PLG [57]), lipid metabolism (complement C3 [58]), autophagy (DCN [59] and RAB1A [60]), or cell differentiation (SERPINF2 [60]). From the present data, very few membrane proteins were predicted, probably because of their very low concentration [14], but also because of their chemical properties, making them difficult to identify via proteomics. However, the current predicted cell surface proteins are consistent with the muscle or AT biology, as they may transduce autocrine or paracrine signals to control tissue growth partly by pathways described previously. Indeed, of the cell surface proteins identified in muscle, ENO1 [61], CAP2 [62], CTSD [63], and PSMC1 [64] were shown to be related to myofiber differentiation; MCAM [65] was shown to be related to myoblast fusion; and COL5A1 [66] was shown to be related to myogenic stem cell commitment. Regarding adipose cell surface proteins, ARRB1 [67], CAPNS1 [68], RPSA [29], and MSN [69] were related to the differentiation of adipose cell precursors. ARRB1 [70] and CTSB [71] were shown to favor the metabolism of brown or beige adipocytes rather than the white adipocytes through PPAR signalling or by regulating mitoautophagy. The complement components C3 and C4A [72] have been proposed to be important in AT maintenance and apoptosis. The adducin (ADD1) [73] was shown to localize atypical protein kinases in 3T3-L1 adipocytes, to signal the insulin receptor, and to cause the translocation of glucose transporters on cell surfaces during differentiation. Either keratin, type II cytoskeletal 1 (KRT1) [74], or Ras-related protein Rab-14 (Rab14) [75] was shown to be involved in the interaction and signalling or intracellular trafficking of the glucose transporter isoform 4 during the processes of adipocyte hypertrophy.

These putative secreted or cell surface proteins may also contribute to the “adipose–muscular” crosstalk through interaction. Among the proteins potentially secreted by AT and having a potential role in the regulation of muscle growth, we highlight an interaction between the adipokine plasminogen (PLG) and the muscular cell surface protein Alpha-enolase (ENO1). ENO1 is mainly described for its role as an intracellular glycolytic enzyme, however its presence as a cell surface protein has been already reported in muscular cells, particularly on the cell surface of differentiating myocytes [61]. High expression of ENO1 during fetal life has been described in mammals and birds, with a shift from α-enolase ENO1 to β-enolase ENO3 late in gestation or during in ovo life [61]. ENO1 was described as a receptor for PLG in several cell types, including skeletal muscle cells, serving to localize and promote plasminogen activation pericellularly. Inhibition of ENO1–plasminogen binding was described to block myogenic fusion in vitro, indicating that ENO1–plasminogen signalling appeared as an important regulator of skeletal myogenesis by concentrating and enhancing plasmin generation on the myogenic cell surface [61]. Of the adipomyokines that may affect the fetal adipogenesis, hemopexin (HPX) may bind the adipose cell surface protein CDC42, and in this way may contribute to the induction and differentiation of adipose stem cells through Wnt–β-Catenin signalling. This putative interaction between CDC42 and HPX is consistent with the observation that HPX knockdown was shown to impair preadipocyte 3T3-L1 differentiation [76]. These data demonstrate that the simultaneous characterization of both the secretome and surfaceome may enhance the understanding of molecular messages embedded in the extracellular environment and involved in autocrine and paracrine regulations.

## 4. Materials and Methods

### 4.1. Muscles and Adipose Tissues Sampling

Fifteen fetuses were generated by artificial insemination of multiparous crossbred beef cows (predominately of Angus breeding) at the North Dakota State University, Beef Research and Teaching Unit (Fargo, ND, USA), as previously described [77]. Fetuses were collected at 140 days post-conception (dpc); and perirenal and omental AT, *Semitendinosus* (ST), and *Longissimus thoracis* (LT) muscles were sampled. Tissues were stored at −80 °C pending protein extraction. All procedures involving animals were approved by the North Dakota State University (NDSU) Animal Care and Use Committee (#A10001).

### 4.2. Large-Scale Proteomic Analysis

Total proteins were extracted as previously described from AT [11] and from muscles [78]. In order to fractionate proteins and to increase the identification of fetal proteins, proteins were separated by two-dimensional gel (2-DE) electrophoresis performed according to Bouley et al. [78]. Briefly, for the isoelectrofocalisation (IEF), proteins were solubilized in a rehydration solution (8 M urea, 1 M thiourea, 0.28% dithiothréitol (DTT), 2% CHAPS (3-[(3-Cholamidopropyl)dimethylammonio]-1-propanesulfonate hydrate), 2% immobilized pH gradient (IPG) buffer pH 4–7, and 0.01% coomassie brilliant blue (CBB) G-250). IPG strips covering a pH range of 5–8 were loaded with 700 μg of proteins and were subjected to IEF (73.5 kVh) in a Multiphor II (Amersham Biosciences, Orsay, France) gel apparatus at 20.5 °C. After completion of the IEF, proteins on the strip were equilibrated for 15 min in a buffer containing 6 M urea, 1% DTT, 30% glycerol, 50 mM Tris base, 2% Sodium dodecyl sulfate (SDS) and DTT, and then for an additional 15 min in the same solution, except DTT was replaced by 5% iodoacetamide. The immobilized pH gradient strips were then transferred onto 12% T, with 2.6% C separating polyacrylamide gels, and proteins were separated in the second dimension using a Protean Plus Dodeca cell system (BioRad, Hercules, CA, USA). Gels were stained with G250 colloidal Comassie Blue. Protein spot detection was realized with SameSpots V4.5 software (Nonlinear Dynamics, Newcastle, UK). Protein spots from the two ATs (30 gels, 15 for perirenal and 15 from omental ATs) or the 2 muscles (30 gels, 15 from *Semitendinosus* and 15 from *Longissimus thoracis* muscles) were matched to a reference gel chosen from the 30 gels from one tissue type. We detected 368 muscular and 359 adipose well-resolved protein spots. The maximum number of protein spots was sampled for identification.

Gels were washed with distilled water and protein spots were excised individually and placed in sterile Eppendorf tubes. Spots were discolored in 100 µL of 25 mM NH4HCO 3–5% acetonitrile buffer over 30 min, and then in 100 µL of 25 mM NH4HCO 3–50% acetonitrile buffer for 30 min twice. Spots were then dehydrated for ten minutes in 200 µL of 100% acetonitrile. Acetonitrile was removed and samples were dried during 10 min using a Speed Vac (Thermo Fisher Scientific, Illkirch-Graffenstaden, France). Proteins were hydrolyzed in peptides using 150 ng of trypsin solution (12 µL of a 25mM NH4HCO3 containing 12.5 ng/µL of typsin). After 5 h of incubation at 37 °C, peptides were extracted using sonication with 9.6 µL of 100% acetonitrile. Supernatants were transferred in High-Performance Liquid Chromatography (HPLC) vials and dried using a Speed Vac for 10 min. Then, 10 µL of equilibration solution (H2O/formic acid-100/0.5) was added before Nanoscale liquid chromatography coupled to tandem mass spectrometry (nano-LC-MS/MS) analysis.

The peptides mixtures were analyzed by nano-LC-MS/MS using an Ultimate 3000 system (Dionex, Voisins le Bretonneux, France) coupled to an LTQ velos (Thermo Fisher Scientific, Illkirch-Graffenstaden, France) mass spectrometer with a nanospray ion source. After injection, 8 µL of peptide mixture was concentrated at a flow rate of 30 µL/min with a trap column (5 cm, 100 µm internal diameter, named Acclaim PepMap 100 C18, 5 µm, 100 A; Thermo Fisher Scientific, Illkirch-Graffenstaden, France) equilibrated with trifluoroacetic acid 0.05% in water. After 6 min of desalting, a valve was switched and the separation of peptides mixture was carried out according to their hydrophobicity with a nanocolumn (Acclaim PepMap 100 C18, 75 µm, 15 cm, (Thermo Fisher Scientific, Illkirch-Graffenstaden, France) equilibrated with 0.5% formic acid in water. The solvent gradient increased linearly from 4% to 90% acetonitrile in 0.5% formic acid at a flow rate of 300 nL/mn for 38 mn. The peptides eluate was electrosprayed in positive-ion mode at 1.6 kV through a nanoelectrospray ion source operated in Collision-induced dissociation top 5 mode (i.e., 1 full MS scan, whereby the 5 major peaks in the full scan were selected for MS/MS). The parameters of mass spectrometry analysis were as follows: full-enhanced-scan MS spectra acquired with 1 microscan (*m*/*z* 400–1400), dynamic exclusion used with 2 repeat counts, 30 s repeat duration, and 60 s exclusion duration. For MS/MS, the isolation width for the ion precursor was fixed at 2 *m*/*z* and single charged species were rejected; fragmentation used 37% normalized collision energy as the default activation of 0.25.

At the end of each LC-MS/MS analysis, the raw files were loaded into the Proteome Discoverer v1.4 (Thermo Fisher Scientific, Illkirch-Graffenstaden, France) and identification was performed with Mascot v2.5 (http://www.matrixscience.com, Matrix Science Ltd, London, UK). The database used for the search was UniProt Bos taurus (37,513 sequences, FASTA file version: 7 November 2019), with the following parameters: peptide mass tolerance was set to 1.5 Da, fragment mass tolerance was set to 0.5 Da, and a maximum of two missed cleavages were allowed. Variable modifications were methionine oxidation (M) and carbamidomethylation (C) of cysteine. In this analysis, a peptide is validated when its Mascot score is greater than 39, which indicates a significant match for the announced sequence (*p*-value < 0.05; false discovery rate (FDR) < 5%). Protein identification was considered valid if at least 3 peptides with a statistically significant Mascot score were assigned it. Criteria of FDR < 5% rather than < 1% plus 3 peptides were chosen to maximize the discovery of adipose and muscular proteins for a large description of the secretome and surfaceome. Proteomics analyses were carried out on the two anatomical sites of adipose tissue or muscle in order “maximize” the discovery of adipose or muscular proteins for the most complete description of the secretome, surfaceome, and the crosstalk between tissues.

### 4.3. Bioinformatic Analysis and Data Mining

**Figure 5:**
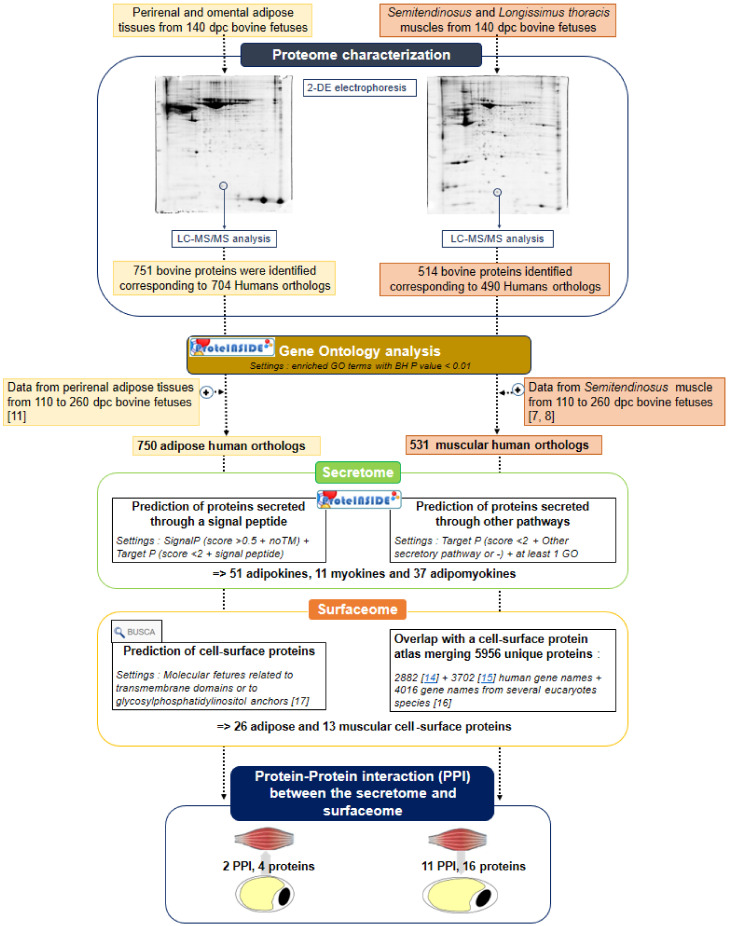
Flowchart of the workflow applied for the discovery of pathways involved in bovine fetal myogenesis and adipogenesis, and for the prediction of the secretome and surfaceome of fetal bovine muscles and adipose tissues, using data from the present proteomic characterization and data from the literature. Settings used for GO analysis to declare a protein as putatively secreted or located at the cell surface are indicated. Abbreviations used: dpc: days post conception, 2-DE: two-dimensional gel electrophoresis, BH: Benjamini–Hochberg adjusted *p*-values, noTM: no transmembrane domain.

Proteomics data were mainly mined with ProteINSIDE [79] (INRAE, Saint-Genès-Champanelle, France; version 1.2/last update 10 April 2020, https://www.proteinside.org). The UniProt identifiers of the bovine proteins identified in adipose and muscular tissues were automatically translated into human identifiers using orthologous and homologous links that are known between the two species and proposed by ProteINSIDE to take advantage of the most complete annotation available for human genes. Indeed, as genes linked by evolution, particularly orthologs, usually have conserved functions between vertebrates and because the human is the most annotated species, human orthologs of bovine proteins were considered in order to further investigate their biological functions. Either human or bovine identifiers were, used depending on the step of the data mining workflow (Figure 5), either to describe the original dataset obtained or to predict the adipose and muscular secretomes, surfaceomes, and the potential crosstalk between tissues.

#### 4.3.1. Gene Ontology (GO) Analysis

Identifiers for adipose and muscular proteins were launched independently for a basic GO analysis (default options within ProteINSIDE, http://www.proteinside.org) in order to identify biological pathways associated with fetal adipogenesis and myogenesis. The GO enrichment analyses were achieved in the human species using human orthologs. The Benjamini–Hochberg (BH) adjusted *p*-values were considered to establish lists of significant enriched pathways in each dataset as compared to the whole genome. Only GO terms with BH *p*-values < 0.01 that had annotated at least 2 proteins were considered to describe the adipose and muscular fetal datasets. Additionally, biological functions related to adipogenesis and myogenesis were searched from results of the IDresume module provided by ProteINSIDE, gathering descriptions of protein functions from Uniprot and NCBI databases.

#### 4.3.2. Creation of a Compendium of Fetal Adipose and Muscular Proteomes

In order to obtain the largest and most meaningful secretome and surfaceome data for fetal bovine tissues, we merged the data acquired in the present study with published data in order to create a compendium. Thus, data from the present fetal proteomes were merged with previously identified fetal muscular [7,8] and adipose [11] proteomes. To date, available data have allowed a list of 750 adipose and 531 muscular unique proteins present in the bovine fetal adipose or muscular tissues, respectively.

#### 4.3.3. Computational Prediction of the Proteins Secreted by Fetal Adipose Tissue or Muscle

ProteINSIDE was used to first predict the presence of a signal peptide on a protein sequence (imported by the biological knowledge retrieval script) through a local version of the SignalP algorithm [80]. The predictions were verified by both GO term annotations related to extracellular components and cellular locations predicted through a local version of TargetP [81]. For this prediction, we used the identifiers and protein sequences from the bovine species that were launched in the “custom analysis” with the choice of the parameter “increase cleavage site sensitivity (D-cutoff 0.34)”. The following criteria were used to declare a protein as putatively secreted through a signalP sequence: non-transmembrane (noTM) domain, SignalP score > 0.5, and TargetP score ≤ 2. The second prediction analysis aimed to identify proteins that are secreted through unconventional pathways (i.e., membrane flip-flop or vesicule secretion [82]), through a local version of TargetP [81] and the use of GO terms related to extracellular components. For this prediction, we used the identifiers and protein sequences from the bovine species that were launched in the “basic analysis” with the default options. The following criteria were used to declare a protein as putatively secreted through pathways that do not involve a SignalP sequence: TargetP annotation “other secretory pathway”, plus TargetP score ≤ 2, plus at least one GO term related to an extracellular location. In order to confirm the results, the prediction of proteins secreted both by a signal peptide or by others pathways was compared to the lists of proteins declared in the human plasma database [18]), of proteins identified in bovine plasma [19], and proteins proposed to be secreted by AT or muscle in bovine [20].

#### 4.3.4. Computational Prediction of the Fetal Adipose Tissue or Muscle Surfaceome

The surfaceome was defined as all plasma membrane proteins that have at least one amino acid residue exposed to the extracellular space [14]. The experimental assessment of the surfaceome is complicated by the very low abundance of cell surface proteins compared to intracellular proteins and the hydrophobic nature of transmembrane domains. Thus, several predictive methods for the surfaceome were developed and some of them were integrated in the BUSCA web server (http://busca.biocomp.unibo.it/; Bologna Biocomputing Group, Bologna, Italy) [17]. The BUSCA web server relies on different machine and deep learning approaches to recognize and predict features that are relevant to determine protein subcellular locations. The first category of methods used by BUSCA are for the surfaceome discovery, identifying molecular features related to transmembrane domains along the input protein sequence. The second category of methods relies on the subcellular location prediction.

We applied two strategies to predict the bovine fetal adipose and muscular surfaceomes. The first one was the prediction of the surfaceome using the BUSCA web service for eukaryota and animals. For this prediction, we used the bovine protein sequences, with the following criteria used to declare a protein as putatively located at the cell surface membrane: the presence of at least one α- helical or β-barrel transmembrane domain or of a glycosylphosphatidylinositol (GPI) anchor plus the GO term annotation GO:0005886 for the plasma membrane. The second strategy aimed to compare the gene names of the 750 adipose and 531 muscular proteins to the gene names of the plasma membrane proteins identified by complementary predictive methods. Thus, we built a compendium of cell membrane proteins by merging 2882 [14] and 3702 [15] human gene names with 4016 gene names from several eukaryotic species [16]. A total of 5956 unique proteins were merged and were used as a reference atlas to compare the fetal bovine proteomes from AT and muscle.

#### 4.3.5. Computational Prediction of Protein–Protein Interactions between Cell Surface Proteins and Secreted Proteins

Interactions between proteins secreted by a tissue and cell surface proteins from another tissue were searched with the Protein–Protein Interactions (PPI) module of ProteINSIDE. PPI between the proteins were identified thank to the PPI referenced within UniProt (https://www.uniprot.org) [83], IntAct (https://www.ebi.ac.uk/intact/main.xhtml) [84], and BioGrid (https://thebiogrid.org) [85] databases. The input data were the gene names of the human orthologs of bovine proteins in order to take advantage of the well-known PPI characterized in humans. As already described [79], these PPI databases were chosen as a default option in the PPI analysis module of ProteINSIDE because these are updated daily and reviewed by curators. Moreover, they are included in curation processes of the IMEx (which ensures reliable interaction data using experts and curation rules shared between many interaction databases [86]) or MIMIx projects (a guideline of the minimum information required for reporting a molecular interaction experiment, thus advising the user on how to use the interaction data [87]). Only PPI that were proven by experiments are considered by ProteINSIDE. Lastly, and to ascertain the biological significance of these interactions in the context of AT and muscle biology, we verified the synthesis of a protein by a tissue by using the experimental results reported in the Expression Atlas database (https://www.ebi.ac.uk/gxa/home) [88].

## 5. Conclusions

To conclude, this study provides new data on the molecular events underpinning simultaneously bovine myogenesis and adipogenesis at a fetal age corresponding to increases in both cell number and volume for AT and muscle. These data are valuable for issues related to the lean-to-fat ratios of bovine carcasses and meat for the beef industry, but also for knowledge relevant to the ontogenesis of human body composition. Indeed, available data revealed that myogenesis in cattle is very close to that of humans (i.e., same types of fibers, same gestation length, similar kinetics of expression of developmental myosin heavy chain isoforms, and total number of fibers set about at the end of second trimester). A large part of the data provided by the present study remains to be explored, and thus are made available for AT and muscle physiologists interested in understanding the fetal growth of these tissues.

## Figures and Tables

**Table 1:** Proteins potentially secreted through a signal peptide by bovine fetal adipose tissue (AT), muscle, or both tissues. The predictions were performed using the Signal P algorithm and were confirmed by the subcellular location provided by TargetP and Uniprot, thanks to the ProteINSIDE webservice.

Adipose Tissue n = 39	Muscle Tissue n = 9	Adipose and Muscle Tissues n = 29
A2M	LTF	ADIPOQ
AFM	CASQ1	AFP
APOA2	DPT	AHSG
C3	P4HA3	AMBP
CFI	LAMA2	APOA1
COL1A2	C1QTNF3	APOH
COL6A2	DSG1	CLEC3B
COLGALT1	P4HA2	CNPY3
CPB2	SRL	COL5A1
DCN		COL6A1
DDOST		CTSB
DNAJB11		ERLIN2
ERP29		FKBP7
FETUB		HPX
FGG		HSPA5
FKBP10		KNG1
FKBP14		LUM
GC		MESD
GGH		OGN
GLB1		OLFML3
GPX3		P4HA1
HSP90B1		P4HB
ITIH3		PDIA3
MFAP4		POSTN
ORM1		SERPINA1
PCOLCE		SERPINF1
PDIA4		THBS1
PLG		TPP1
PLOD3		TTR
PRCP		
RCN3		
RPN1		
SERPINA3		
SERPINC1		
SERPIND1		
SERPINF2		
SERPING1		
SERPINH1		
TGFBR3		

**Table 2:** Proteins potentially secreted by bovine fetal AT, muscle, or both tissues and by pathways that do not involve a signal peptide. The predictions were performed using the TargetP algorithm and were confirmed by the subcellular location provided by Uniprot and at least one GO term related to the secretion, as allowed by the ProteINSIDE webservice.

Adipose Tissue n = 12	Muscle Tissue n = 2	Adipose and Muscle Tissues n = 8
ANP32A	PACSIN3	ANXA1
APPL2	SEPT4	ANXA2
ARRB1		ANXA4
CKAP4		ANXA7
CYB5A		PARK7
EMC2		SNX5
HNRNPA2B1		TPT1
LAMTOR1		VCP
RAB14		
SPET5		
STAM		
TKT		

## Supplementary Materials

Supplementary materials can be found at https://www.mdpi.com/1422-0067/21/12/4375/s1.

## Abbreviations

AT	Adipose tissue
Da	Dalton
dpc	Days post-conception
GO	Gene Ontology
ID	Identifier
IEF	Isoelectrofocalisation
PPI	Protein–protein interaction
